# Impact of Mannose-Binding Protein Gene Polymorphisms in Omani Sickle Cell Disease Patients

**DOI:** 10.4084/MJHID.2016.013

**Published:** 2016-02-15

**Authors:** Mathew Zachariah, Shoaib Al Zadjali, Wafa Bashir, Rahma Al Ambusaidi, Rhea Misquith, Yasser Wali, Anil Pathare

**Affiliations:** 1 Departments of Child Health, Sultan Qaboos University Hospital, Muscat, Oman; 2 Departments of Haematology, Sultan Qaboos University Hospital, Muscat, Oman

## Abstract

**Objectives:**

Our aim was to study mannose-binding protein (MBP) polymorphisms in exonic and promoter region and correlate it with associated infections and vasoocculsive (VOC) episodes in sickle cell disease (SCD) patients since MBP plays an important role in innate immunity by activating the complement system.

**Methods:**

We studied the genetic polymorphisms in the Exon 1 (alleles A/O) and promoter region (alleles Y/X; H/L, P/Q) of the *MBL2* gene, in SCD patients as an increased incidence of infections is seen in these patients. A PCR-based, targeted genomic DNA sequencing of MBL2 was used to study 68 SCD Omani patients and 44 controls (healthy voluntary blood donors).

**Results:**

In SCD patients, the frequency of the genotype related to the high production of MBL was 0.35 (YA/YA) and for intermediate/low production was 0.65 (YA/XA, XA/XA, YA/YO, XA/YO, YO/YO). The observed frequencies of *MBL2* gene promoter polymorphism (-221, Y/X) were 44.4% and 20.5% for the heterozygous genotype Y/X and 3.2% and 2.2% for the homozygous (X/X) respectively between SCD patients and controls. *MBL2* Exon1 gene mutations were 29.4% and 50% for the heterozygous genotype A/O and 5.9% and 6.8% respectively for the homozygous (O/O) genotype between SCD patients and controls. The distribution of variant *MBL2* gene polymorphisms did not show any correlation in SCD patients with or without VOC attacks (p=0.16; OR −0.486; CI=0.177 −1.33), however, it was correlated with infections (p=0.0162; OR −3.55; CI 1.25–10.04).

**Conclusions:**

Although the frequency of the genotypes and haplotypes of MBL2 in SCD patients did not differ from controls, overall in the SCD patient cohort the increased representation of variant alleles was significantly correlated with infections (p<0.05). However, these variant MBL2 polymorphisms did not seem to play a significant role in the VOC episodes in this SCD cohort.

## Introduction

Sickle cell disease (SCD) is characterized by a striking variability in the clinical presentation ranging from an early-onset life-threatening disease to a milder condition compatible with an almost normal life course. Polymerization of deoxyHb S with red blood cell (RBC) deformation, desiccation and increased rigidity results in painful vasoocclusive crises (VOC) and hemolytic anemia. Adherence of sickle RBC stimulates endothelial cells to upregulate their adhesion molecules, which accelerates the adhesion cascade.^1^ Activated endothelium also releases a broad range of cytokines, including granulocyte-macrophage-colony-stimulating factor (GM-CSF), interleukin (IL)-1, IL-3, IL-6, IL-8 and tumor necrosis factor (TNF-a), and these have been detected in the plasma of patients with SCD.^2^,^3^ Neutrophils may also become activated during this cascade of vasoocclusive events, and neutrophil adherence may contribute to vasoocclusion,^3^ as well as endothelial cell damage.^4^

Patients with SCD have an increased tendency to infection^5^, especially with encapsulated organisms, which is due in part to the poor splenic function^6^, but might also be a feature of altered neutrophil and monocyte function.^7^ There is now ample evidence indicative of an ongoing inflammatory state between painful crises in SCD involving neutrophils, monocyte activation and an abnormality of cytokine-regulated neutrophil function, which may compromise the host defenses against certain microorganisms.^8^ In this context, polymorphism of the mannose-binding lectin (MBL) has been documented as potential immunogenetic modulating factors that could constitute an additional risk of infection in SCD.^9^ However, polymorphisms of the Fc receptor may, in fact, protect SCD patients from infections with *H. influenzae*.^10^

Mannose-binding lectin (MBL) is a serum protein of hepatic origin belonging to a family of Ca^2+^-dependent collagenous lectins, most of which are components of the innate immune system or natural immunity.^11^,^12^ Mutations in the mannose binding protein gene have been associated with recurrent infections.^13^–^15^ A single gene, *MBL2*, located on chromosome 10, codes for human MBL.^16^,^17^ Mannose-binding lectin may exert its action through binding to mannose-rich, and N-acetyl-glucosamine oligosaccharides present on a variety of microorganisms. Therefore, it activates the complement system by MBL-associated serine proteases and by interacting with novel receptors on phagocytes.^18^–^20^

The mannose binding protein, being part of the innate immune system, is considered particularly important in the vulnerable period of infancy before an adequate specific immune protection was attained by the adaptive immune system.^21^

Five single-nucleotide polymorphisms influencing serum MBL levels have been identified^22^. Three variant alleles have been described in exon 1 of the *MBL2* gene.^23^–^25^ These variants are due to 3 single-base pair substitutions at codon 54 (allele *B*), codon 57 (allele *C*), and codon 52 (allele *D*). They, independently, cause low serum MBL levels.^23^ The normal wild type allele is commonly designated *A*, and the three mutant alleles *O*. All variant alleles reduce the amount of functional MBL subunits in heterozygous individuals 5-to 10-fold.^26^ The serum MBL concentration is also dependent on some nucleotide substitutions in the promoter region of the *MBL2* gene.^27^,^28^ In particular, a polymorphism in codon -221 (*X/Y* type) has a significant effect on the MBL serum concentration with the *Y* promoter having high and the *X* having low MBL-expressing activity.^26^–^28^

Given the relatively high prevalence of SCD in the Omani population,^29^ we decided to study the genetic polymorphism of MBL in children and adolescent patients with SCD. The aim of the study is to establish the MBL genotypes in the SCD patients as well as in the Omani ethnic population. The study also attempted clinical correlation with the type, severity of infections and complication seen in SCD patients like VOC’s.

## Materials and Methods

### Patients

The study was conducted on 68 Omani SCD patients, aged between 3–18 years (mean age ± SD; 9.4± 3.9; M:F 37:31), who were enrolled into this case-control study. They were treated at the Pediatric Hematology Unit, Department of Child Health, Sultan Qaboos University Hospital (SQUH). Forty-four ethnically matched healthy voluntary blood donors were included in this study as a control group. The healthy controls were ethnic Omani subjects who were volunteer blood donors aged between 21–44 years (mean age ± SD; 26.4± 2.5; M:F 31:13). They were screened with a CBC and HPLC to confirm that they were indeed normal and then their DNA was extracted for MBP polymorphism study. This study was undertaken after approval by the institutional research and ethics committee and written informed consents were obtained from the patients, guardians in case of minor patients and controls before enrollment. A thorough history and comprehensive examination were conducted with particular emphasis on infections, history of vasooclusive crises and SCD complications. The diagnosis of SCD was confirmed by molecular studies in all the patients to characterize the SCD subtypes and haplotypes. Amongst the 68 SCD patients, 56 patients (82%) were HbSS, 11(17%) were Sickle Thal double heterozygotes, and 1(1%) was Sickle HbD [SD] double heterozygote. All the clinical and laboratory details were obtained from the electronic medical file records in all patients. Patients with infection symptoms were all investigated to document a microbiologically blood culture/ urine culture proven infection.

### DNA studies

A 5ml blood sample was collected in tubes containing EDTA. Genomic DNA was isolated using the semi-automated ABI PRISM 6100 Nucleic Acid Prep Station, [Applied Biosystems, Foster City, CA, USA] and samples were stored at −20°C pending analysis. All the DNA polymorphisms were studied by direct sequencing of the relevant PCR- amplified genome segment on ABI PRISM 3100 Genetic Analyzer (Applied Biosystems, Foster City, CA, USA).

#### Genotyping of Exon 1 of MBL2 gene

Genotyping of exon1 (codon 52, 54 and 57 for alleles D, B, C respectively) was done by multiplex PCR as previously described.^30^

#### Genotyping of the promoter region of MBL2 gene

Genotyping of the promoter region was performed by direct sequencing of the corresponding region of interest (-65 for P/Q alleles, -221 for X/Y alleles and -618 for H/L alleles) by appropriate primers.^31^

#### Analysis of haplotypes and genotypes

Haplotypes of *MBL2* gene were divided into three groups according to Garrett et al.^32^

### Statistical Methods

Data was analyzed using STATA ver. 11.1 (StataCorp, College Station, TX, USA). Numerical data were expressed as mean, standard deviation, range. Qualitative data were expressed as frequency and percentage. Chi-square was used to study the statistical significance of qualitative variables. Fisher’s exact test was used with Yates correction wherever applicable. Odds ratio [OR] and 95% confidence intervals [CI] were calculated for risk estimation. A two-sided p value of less than 0.05 was considered as statistically significant. The observed and expected genotype frequencies were analyzed by using weighted least square estimates of allele frequencies and chi-square goodness-of-fit test to see if Hardy-Weinberg’s proportions were respected.

## Results

The study initially had recruited 85 SCD patients and 50 voluntary blood donor controls. However, as DNA results were available only in 68 SCD patients and 44 normal controls, this was the study population analyzed in further details and reported herein. There were 37 males and 31 females in the patient group. Their age ranged from 3 to 18 years with the mean age of 9.4 years.

The results of genotype and allele frequencies of the Exon-1 and promoter *MBL2* gene polymorphisms are shown in Table 1. MBP exon1 and promoter variants were grouped into haplotypes, and all the possible groupings of the observed alleles A/O (B, C, D), H/L, Y/X and P/Q, combined, homozygote and heterozygote, with SCD and no SCD were analyzed and tabulated. In the patients with SCD the MBL2 exon-1 allele was significantly higher while the promoter alleles Q and Y were lower than in controls, (p <0.05). Furthermore, the combined genotype YA/YO was also down represented in the SCD affected, whereas, the minor mutant alleles (O, X and Q) did not obey Hardy Weinberg’s rule.

SCD patients were classified into three categories based on the clinical severity index according to our previous studies.^33^,^34^ Specifically, sickle cell disease patients were categorized as having a mild, moderate, or severe systemic disease based on the history of admissions/year for VOC events and associated clinical SCD complications like Acute chest syndrome, Osteonecrosis, Splenic sequestration, Stroke, Priapism and repeated infections. Patients with more than three hospital admissions/year and/or SCD complications named above were severe cases whereas those with less than one hospital admission for VOC/year were mild, and those, needing between 1–3 admissions/year and SCD complications, were moderate cases.

Table 2 shows the distribution of *MBL2* exon-1 A allele and the mutant allele O and significance of the correlation between the MBL2 polymorphisms and presence or absence of VOC or infections in this cohort of SCD patients. The homozygous or heterozygous mutants [OO+AO] were less common in SCD patients with or without VOC, but this difference was not statistically significant. However, they were more frequent in SCD patients with infections, and the difference was statistically significant (p<0.05).

Of 25 patients (37%), suspected to have an associated infection, 11(44%) had a microbiologically had blood or urine documented culture. In this group of 11 SCD patients, 6 had positive blood cultures (3-Gram positive cocci, and one each with *Bacillus spp*., *E.coli* and *Achromobacter spp*.) The remaining 5 SCD patients had positive urine cultures (3-*Klebsiella pneumoniae* and 2-*E. coli*).

## Discussion

The MBL2 gene is highly polymorphic and shows allelic variations in different ethnic groups^25^. MBL, the product of *MBL2* gene is an important component of innate immunity, and functionally significant mutations decrease serum MBL levels 18,19. In the current study, MBL2 allelic frequencies were not significantly different in the SCD group as compared to the control subjects, especially the clinically relevant MBL2 expression status (high, intermediate and low; Table 1). Although small differences were observed in the Egyptian and Brazilian SCD patients, in both these studies, like our study, there were no statistically significant allelic differences seen between controls and SCD patients.^35^,^36^

Our study, however, found an evident association between MBL2 variants with infection with an odds ratio of 3.55 (Table 2). This observation is in accordance with several other previous studies.^9^,^11^ Neonato et al. (1999) reported that mutations in MBL2, which were associated with low levels of MBL, showed increased susceptibility to infections in their cohort of SCD patients.^9^ MBL is probably involved in the recognition of sickle RBC’s and removing them from the site of inflammation.^9^ However with low MBL levels in patients with mutant alleles; this process is hampered. Thus, although MBL is known to be associated with innate immunity, the presence of variant *MBL2* genes is considered to affect the efficiency of complement-phagocyte interaction leading to an increased propensity for infections, especially of encapsulated microorganisms as suggested by increased pneumococcal infections.^37^ This interpretation is also corroborated by the routine administration of the pneumococcal vaccine in SCD children below five years inducing a decrease of pneumococcal infections.^38^ Unfortunately, in this study we were unable to measure the serum MBL levels, although it was planned as part of this study, due to certain technical shortcomings.

Curiously, a couple of studies have reported the association of MBL variant genes with vasooclusive crisis from Brazilian SCD patients. Oliveira et al. (2009) and Mendonca et al. (2010) reported that there was an association between MBL2 structural polymorphism with VOC.^36^,^39^ In our study, we did not find any association between MBL2 variants with VOC (Table 2). This difference is due to the characterization of VOC events, which was quite different. In both these studies from Brazil, the presence of only one VOC was used as an index of SCD severity, whereas, in our study we have classified patients as severe with three or more than three VOC events/year, and mild with one event.^33^,^34^ This classification is recommended by the Cooperative Study of Sickle Cell Disease (CSSCD).^40^

Furthermore, SCD patients from Oman are a mixture of several African and Asian β^s^ haplotypes with 52.1% showing Benin haplotypes, 26.7% showing Arab-Indian haplotypes, and 21.4% showing Bantu haplotypes.^41^ It is thus apparent that almost two-thirds of these patients have a severe form of disease and hence have a higher severity index.^33^,^34^ VOC’s are a complex process with an interaction between sickle reticulocytes, perturbed endothelium, and inflammatory cytokines. It is thus hard to mechanistically understand how such complex interactions could be affected by MBL2 polymorphisms which as stated above are well correlated to susceptibility to infections.

Oman has shown a remarkable progress in several fields like education, health care and socio-economic progress in the last several decades. However, autosomal recessive disorders like SCD persist in the population due to the practice of consanguineous marriages.^42^ Thus, there is quite a disparity in the observed and expected frequency of minor MBL2 alleles X, O, and Q where the Hardy-Weinberg equation is not obeyed (Table 1). Nevertheless, it is certain that MBL2 variant alleles are significantly associated with infections in SCD patients from Oman.

## Figures and Tables

**Table 1:** Genotypic and allele frequencies of *MBL2* gene in Omani SCD patients and controls.

	SCD patients, n(%)	Controls, n(%)	P value
Exon 1			
A/A	44/68(64.7)	19/44(43.2)	0.227
A/O	20/68(29.4)	22/44(50.0)	0.144
A/B	6/68(8.8)	10/44(22.7)	0.078
A/C	7/68(10.3)	7/44(15.9)	0.44
A/D	7/68(10.3)	5/44(11.4)	0.87
O/O	4/68(5.9)	3/44(6.8)	0.85
C/C	--	2/44(4.5)	0.313
B/B	4/68(5.9)	--	
B/C	--	1/44(2.3)	0.216
Allele Frequency(HW frequencies)			
A	0.647(0.63)	0.432(0.465)	<0.0001*
O	0.059(0.042)#	0.068(0.101)#	0.426
Promoter Region[−289C>G](%)			
Y/Y	33/63(52.4)	34/44(77.3)	0.214
X/Y	28/63(44.4)	9/44(20.5)	0.067
X/X	2/63(3.2)	1/44(2.2)	0.786
Allele Frequency(HW frequencies)			
X	0.0317(0.0645)#	0.023(0.016)#	0.225
Y	0.524(0.557)	0.773(0.766)	<0.0001*
Promoter [−65C>T](% )			
P/P	34/63(53.9)	22/44(50.0)	0.82
P/Q	28/63(44.4)	17/44(38.6)	0.7
Q/Q	1/63(1.7)	5/44(11.4)	0.042*
Allele Frequency(HW frequencies)			
P	0.54(0.581)	0.5(0.48)	0.696
Q	0.016(0.057)#	0.114(0.84)#	<0.0001*
Combined Genotypes(%)			
YA/YA	22/63(34.9)	11/44(25.0)	0.423
YA/XA	17/63(26.9)	8/44(18.2)	0.4
YA/YO	9/63(14.3)	20/44(45.5)	0.007*
YA/XO	8/63(12.7)	2/44(4.5)	0.19
XA/XA	2/63(3.2)	0	0.24
YO/XA	1/63(1.7)	0	0.404
YO/YO	2/63(3.2)	3/44(6.8)	0.403
YO/XO	2/63(3.2)	0	0.24
MBL2 Expression status(%)			
High(YA/YA)	22/63(34.9)	11/44(25.0)	0.423
Intermediate(YA/XA,XA/XA,YA/YO)	28/63(44.4)	28/44(63.6)	0.278
Low (Other combinations)	13/63(20.6)	5/44(11.4)	0.166

* P value <0.05, Chi square test;

# –Discordant Hardy-Weinberg

**Table 2:** Correlation of MBL2 Exon 1 variant polymorphism with VOC and Infections in the SCD patient cohort.

Symptoms	MBL2 Exon 1 polymorphisms		Total	P value	Odds Ratio	Confidence Interval
	AO+OO	AA				
VOC						
Yes	10	25	35	0.16	0.48	0.177 – 1.33
No	14	17	31			
Infections						
Yes	13	11	24	0.0162*	3.55	1.25–10.04
No	11	33	44			

* P value <0.05, Chi square test
